# Barriers and facilitators of ongoing engagement in substance use treatment programmes among people with HIV in South Africa: A qualitative analysis

**DOI:** 10.4102/sajhivmed.v27i1.1776

**Published:** 2026-02-23

**Authors:** Thanusha Pillay, Stefani Du Toit, Chiara Sforza, Abigail Hines, Alexandra Rose, Jessica Magidson, John Joska

**Affiliations:** 1 Department of Psychiatry and Mental Health, Faculty of Health Sciences, University of Cape Town, Cape Town, South Africa; 2 HIV Mental Health Research Unit, Division of Neuropsychiatry, Department of Psychiatry and Mental Health, Faculty of Health Sciences, University of Cape Town, Cape Town, South Africa; 3 Department of Psychology, University of Maryland, College Park, Maryland, United States; 4 The Global Mental Health and Addiction Program, University of Maryland, College Park, Maryland, United States; 5 Department of Psychiatry and Behavioral Sciences, University of Washington, Seattle, Washington, United States; 6 Department of Psychology, Center for Substance Use, Addiction and Health Research, University of Maryland, College Park, Maryland, United States

**Keywords:** HIV, substance use, treatment engagement, qualitative research, South Africa

## Abstract

**Background:**

South Africa has a high prevalence of HIV and substance use, with 13% of the population meeting criteria for lifetime substance use disorder (SUD). Substance disorders are associated with adverse health outcomes, including poor adherence to antiretroviral therapy (ART), reduced health-seeking behaviour and increased risk-taking behaviour. Limited research has explored the perspectives of people with HIV in low- and middle-income countries when accessing substance use treatment.

**Objectives:**

To identify barriers and facilitators influencing engagement in substance use treatment programmes among people with HIV and SUDs, drawing on perspectives from both patients and healthcare providers.

**Method:**

This qualitative study analysed individual interviews from Project Khanya, a peer-delivered intervention promoting ART adherence among adults living with HIV with at least a moderate SUD. Using criterion sampling, 34 adults with HIV and nine healthcare providers involved in HIV or substance use care were included. Inductive thematic analysis of transcripts from audio recordings identified individual, social, and structural factors influencing treatment engagement.

**Results:**

Participants had a mean age of 39.2 years and 48% were female; providers had a mean age of 40.8 years and were predominantly female. Five main subthemes influenced engagement in substance use treatment: social support, substance use, service-related factors, readiness to change, and financial constraints. Facilitators included strong social support, positive healthcare experiences, outreach, psychoeducation, insight, and reduced substance use, while barriers included poor support, stigma, negative healthcare interactions, financial challenges, and ongoing substance use.

**Conclusion:**

Intersectional stigma, privacy concerns, and ongoing substance use hinder engagement in HIV and SUD treatment, whereas holistic, person-centred approaches can improve programme attendance and health outcomes.

**What this study adds:** This study adds insight into the intertwined individual, social, and structural factors shaping engagement in substance use treatment among people with HIV in South Africa. By integrating perspectives from both patients and providers, it highlights the need for holistic, person-centred, and stigma-sensitive approaches to improve treatment adherence and health outcomes.

## Introduction

South Africa has an HIV prevalence of 14%, with even higher rates in peri-urban areas.^1,2,3^ A syndemic of HIV and substance use is evident in low socioeconomic settings, which are characterised by constrained healthcare access, high population density, and elevated levels of substance use.^4,5,6^

Substance use disorders (SUDs) affect approximately 13.3% of the population in South Africa; the Western Cape province has the highest prevalence of substance use at 20.6%.^3,7,8^ Substance use disorders, including illicit substances and alcohol, can be screened for using the WHO–ASSIST (World Health Organization – the Alcohol, Smoking and Substance Involvement Screening Test), which categorises the risk as low, moderate and severe according the scoring system.^9^ SUD is associated with significant social and health consequences including high-risk behaviours that increase HIV transmission risk, such as sharing needles, transactional sex, non-consensual sexual intercourse and higher rates of unprotected sex, resulting in higher incidence of HIV.^10,11,12^ The initiation of substance use can often follow HIV diagnosis, reflecting the challenges of coping with fear of death and denial.^13^ Individuals may engage in substance use to reduce psychological discomfort, despite negative effects on long-term health outcomes.^11^

In low- and middle-income countries (LMICs), fewer public substance use treatment programmes are available, compared with high-income countries.^14^ Barriers to attendance of available substance use treatment programmes include poor care integration, limited community involvement, stigma, and social determinants such as poverty, limited education, and transport costs.^6,11,15,16,17,18,19,20^ In South Africa, as in other LMICs, qualified professionals and substance use services are available in both the public and private sectors. These resources are predominantly concentrated in higher socioeconomic areas, limiting access for lower-income communities.^11,14^ The current issues include limited evidence for and funding of peer-delivered intervention models, which restrict training opportunities and task sharing, particularly in LMICs for people with HIV and comorbid substance use.^17,21^

Engagement in substance use treatment can be facilitated by a strong motivation for change, heightened awareness of available treatment options, and support from community healthcare providers (HCPs).^12,22^ Furthermore, strategies such as integrated care, decentralised service delivery, community-based outreach, and the provision of culturally tailored services have been shown to enhance both access to and retention in care.^15,17,18,23^

People with HIV and SUD face unique barriers including intersectional stigma, which arises from the interrelated and synergistic effects of these two conditions.^24^ These barriers contribute to poorer HIV care outcomes, including lower adherence to antiretroviral therapy (ART), delayed initiation, missed appointments, and reduced viral suppression.^17,25,26^

The current literature focuses on high-income countries, leaving gaps in knowledge about LMICs.^23^ The context of LMICs, with their limited resources, poverty, and diverse psychosocial needs, requires locally adapted solutions to improve engagement in substance use treatment programmes.^16^ Given the high prevalence of both HIV and SUDs in South Africa and their syndemic relationship, it is crucial to develop appropriate interventions to improve health outcomes.

This qualitative study investigated the barriers and facilitators to substance use treatment from the perspectives of both patients and healthcare practitioners (HCPs) involved in a pilot integrated intervention. While prior research has often examined these perspectives separately, few studies have integrated points of view of patient and provider in a single analysis. By doing so, this study provides a unique and comprehensive understanding of the multi-level factors influencing access to and engagement with substance use care. Importantly, it highlights how the experiences of patients intersect with provider practices, offering insights into both individual and systemic dynamics that shape treatment outcomes. The study aims to bridge the gap in literature and to identify whether the known literature is relevant in a peri-urban South African primary healthcare setting, or whether additional context-specific factors are at play.

## Research methods and design

### Study design and setting

This qualitative study draws on one-off, semi-structured interviews collected from a larger study, ‘Project Khanya’, implemented from 2018 to 2019 in Khayelitsha, a peri-urban area of Cape Town.^27,28^ Project Khanya refers to a time-limited, peer-delivered study, which incorporated behavioural activation, problem-solving therapy, motivational interviewing, and mindfulness-based relapse prevention to support substance use cessation and to improve ART adherence.^10^ The pilot study included the collection of quantitative and qualitative data.^27,28^ The post-study qualitative interviews represented a unique opportunity to obtain a direct account from HCPs and Project Khanya patient-participants, in order to determine potential barriers and facilitators to ongoing engagement in substance use treatment programmes. This was the focus of the present study.

### Study population and sampling

Participants were purposively sampled, using criterion sampling, by HCPs from a primary healthcare facility for study eligibility in Cape Town.^27,28^ Participants were eligible for inclusion if they were HIV positive, aged 18–65, and scored at least moderate for substance use on the WHO-ASSIST (score ≥ 11 for alcohol or ≥ 4 for other drugs) with a history of ART non-adherence: defined as missing one or more pharmacy refill dates, receiving second-line treatment, requiring re-initiation on first-line ARTs, or having a detectable viral load (≥ 400 copies/mL).^9,27,28,29^ Participants were excluded if they had harmful opiate or alcohol dependence requiring medical intervention, an untreated comorbid mental health condition, were currently enrolled in a substance use treatment programme, were unable to provide informed consent, were unable to communicate in English or isiXhosa, or were in the third trimester of pregnancy.^28^

Originally, 66 individuals were enrolled; however, five skipped the follow-up, resulting in a final sample of 61 participants. Of these, 30 were assigned to the Project Khanya intervention, and 31 were allocated to the enhanced treatment-as-usual (ETAU) group. Participants in the ETAU group did not receive the Project Khanya intervention but were instead provided with a referral letter to the Matrix programme, a government-subsidised and widely accessible substance use treatment service in the Western Cape.^28,30^ Referral support from Project Khanya staff included discussing the referral with participants, offering accompaniment to the initial intake appointment, and conducting follow-up regarding subsequent visits.

In total, 43 transcripts were available for analysis – consisting of transcripts from the participants who underwent the Project Khanya intervention (*n* = 23 [*N* = 30]; 76.76%), ETAU participants (*n* = 11 [*N* = 31]; 35.48%) and transcripts from HCPs (*n* = 9).^28^ Of the 30 participants in the Project Khanya intervention group, 23 interviews were available for analysis.^28^ Two participants had died, three could not be reached, and two interviews were excluded because of poor audio quality. In the ETAU group, only 11 participant interviews were available for analysis. In total, 34 participant transcripts were analysed.

In addition, nine interviews were conducted with HCPs, including nurses, substance use counsellors, adherence counsellors, supervisors, and administrators. These providers were involved directly in the delivery of HIV and substance use services in primary care clinics or co-located treatment programmes.^27^

### Data collection

Participant and HCP interview guides were developed iteratively to reflect the objectives of the study (Online Appendix 1). The participant interview guide explored substance use behaviours, ART adherence, and perceived barriers and facilitators to engaging in substance use treatment. The HCP interview guide explored perspectives on the intervention, including its influence on patient outcomes, structural and clinical barriers and facilitators influencing the access to and engagement of patients in treatment. For example, HCPs were asked about strategies used to enhance early treatment engagement (e.g. ‘What strategies do you or your colleagues use to enhance patient engagement and attendance at Matrix, particularly during the initial stages of treatment?’). Open-ended prompts (e.g. ‘Is there a theme related to substance use or treatment adherence that you believe should have been incorporated into the intervention?’) were included to elicit candid and nuanced reflections and to allow participants to raise issues not captured in structured questions.

All the interviews were conducted by a trained and supervised research assistant who was fluent in both English and isiXhosa. All interviews were recorded and transcribed. Quality control involved verification of isiXhosa audio against English transcripts by a fluent isiXhosa-speaking assistant.

Although the transcripts included information beyond the scope of the current study, only the selected information pertaining to the barriers and facilitators to ongoing engagement in substance use treatment programmes was selected for analysis.

### Data analysis

In total, 43 transcripts were available for analysis. The transcripts were reviewed to ensure completeness before analysis. Thematic analysis was guided by the six steps outlined by Braun and Clarke.^31^ Transcripts were imported into NVivo v14 for inductive coding by a primary coder (TP), who independently developed a preliminary codebook of 34 codes.^32^

Although these codes aligned broadly with individual, social, and structural factors commonly described in the literature, the analysis remained inductive to ensure sensitivity to locally relevant content. The secondary coder (CS) and a reviewer (SDT) reviewed and refined the codebook for clarity and coherence. Both coders jointly coded an initial set of HCP transcripts to establish consistency. Owing to time constraints, the finalised codebook was used by both coders throughout the coding process. The secondary coder independently coded a randomly selected sample from both participant groups. Coders met regularly to discuss interpretations and to maintain coding reliability.

The double coding process helped to ensure coding consistency, analytical rigour, and the identification of key quotations for theme development. Data saturation was achieved for Project Khanya participants, while the smaller ETAU and healthcare-provider samples offered more limited perspectives, in line with CORE-Q guidance.^33,34^

All codes and representative quotations were reviewed by the reviewer (SDT) with discrepancies resolved through discussion. Overarching themes were organised into subthemes inductively, without predetermined categories, to reflect locally relevant factors.

### Reflexivity statement

Both coders have healthcare backgrounds: one is a US-based medical student and the other a South Africa-based healthcare worker experienced in HIV and substance use care. Their differing contexts probably influenced interpretation, mitigated through ongoing discussion, clarification of contextual terminology, and supervisory review by a third researcher. Double-coding, supervisory oversight and transparent documentation aimed to enhance analytical rigour and trustworthiness.

### Ethical considerations

Ethical clearance to conduct this study was obtained from the University of Cape Town, Faculty of Sciences Human Research Ethics Committee (No. 495/2024).

## Results

The demographics of the healthcare worker participants and patient participants are noted in Table 1.

**TABLE 1:** Demographics of the participants and providers.

Demographics	Project Khanya participants (*n* = 23)	ETAU participants (*n* = 10)	Healthcare worker participants (*n* = 9)
Average age (years)	39.8	34.3	40.8
Female (%)	43.3	64.5	56.0

Source: Magidson JF, Joska JA, Belus JM, et al. Project Khanya: results from a pilot randomized type 1 hybrid effectiveness-implementation trial of a peer-delivered behavioural intervention for ART adherence and substance use in HIV care in South Africa. J Int. AIDS Soc. 2021;24:e25720

ETAU, enhanced treatment as usual.

Five main subthemes emerged as barriers or facilitators for ongoing treatment engagement: social support, substance use, service factors, readiness to change, and financial constraints.

### Social support

#### Support as a facilitator of treatment engagement

Families, partners, and community members play a critical role in promoting engagement in care, adherence to treatment, and efforts to reduce substance use. Participants emphasised consistently that these support networks were essential in providing both practical assistance and motivational encouragement to initiate and sustain involvement in treatment and ongoing care. For instance, one ETAU participant noted their parents’ encouragement:

‘They really encouraged me a lot, especially when I’ve received a call [*from the clinic*] … also [*provide*] the transport money to attend the clinic.’ (ETAU 1007)

Healthcare providers similarly emphasised the need to involve family members in interventions, noting that doing so could enhance support, reduce stigma, and improve continuity of care. The HCP stated:

‘[*We*] need to call the families and educate them, involve them in the intervention so that the patient can see that they are not on their own.’ (HCP 03)

Another HCP added:

‘[*W*]hatever treatment modality, you have to … involve their family members. They are people who can support the person while, while undergoing this treatment or intervention.’ (HCP 03)

#### Social environments as barriers to care

Participant and provider narratives highlighted that social environments could also undermine treatment. Peer pressure, maltreatment from support systems and community norms often encouraged substance use and undermined treatment adherence. One KP described:

‘[*Y*]ou get pressured [*by community members*] into drinking alcohol even if you don’t want to drink alcohol.’ (KP 1037)

Another KP noted:

‘[*B*]eing with my friends most of the time has made me to drink a lot.’ (KP 1025)

A HCP echoed these concerns:

‘[*Community members*] will drive them [*patients*] back to using alcohol and end up not taking their treatment.’ (HCP 01)

#### Stigma and interpersonal stressors

Stigma from both community members and family discouraged care-seeking and often triggered substance use. An ETAU participant described this:

‘[*Community members*] used to swear at me because I’m HIV positive.’ (ETAU 1004)

A HCP similarly noted:

‘[*Because of*] stigma and lack of community support, people may turn to alcohol and drugs as a way to cope … people could be stigmatised; they could be given names … [*They*] may be demotivated by others.’ (HCP 4)

One ETAU participant reported that they used substances because of interpersonal problems with their family:

‘The stresses … made it very difficult for me to easily change or to adapt to this change [*abstinence from substance use*].’ (ETAU 1055).

### Effects of substance use on engagement in care

#### Substance use as a barrier to adherence

Participants identified substance use – driven by daily stressors including stigma, financial hardship, and family responsibilities – as a major barrier to treatment. It had a negative effect on their relationships, adherence to medication, and healthcare engagement, often leading to missed appointments and non-adherence to ART because of forgetting, intoxication, withdrawal, or misinformation.

A HCP observed that the direct effects of alcohol hindered their adherence to their appointment dates:

‘It could be that someone might go to bed drunk, and therefore it becomes difficult for them to wake up and attend sessions.’ (HCP 08)

Another HCP confirmed:

‘[*W*]hen people are drinking, they forget to take their treatment [*antiretroviral medication*].’ (HCP 02)

An ETAU participant echoed this sentiment:

‘[*W*]hen I’ve drank alcohol, I would not take my medication.’ (ETAU 1019)

Conversely, an ETAU participant noted that reduction of substance use increased their adherence to treatment, explaining:

‘I managed to reduce my drinking, and I was able to take my medication.’ (ETAU 1042)

#### Health concerns as a catalyst for change

Some participants expressed concerns about the physical consequences of not adhering to ART and continued substance use. One ETAU participant feared developing a mental health care complication owing to substance use:

‘Substance use have made them [*community members*] to have mental problems, so when I see them I think to myself … maybe this could have been me.’ (ETAU 1065)

### Service factors

#### Outreach, integration and accessibility

HCPs suggested outreach programmes and community workers to assist in supporting people with HIV and comorbid substance use. The support was noted to relieve some pressure off the staff at the healthcare facilities. One HCP reported that:

‘[*T*]he community care workers, where health workers visit them, support them [*assist with improved engagement in care*].’ (HCP 01)

Other HCPs were in support of outreach programmes:

‘It would be good if it would all be done at the homes, the home of the participant, and even also their community centre. The client would even be more aware of the importance of the study.’ (HCP 02)

Integrated care models were viewed favourably by HCPs, who believed that combining HIV and substance use treatment could reduce stigma and enhance service delivery. One HCP reported:

‘[*I*]t is so important for us to make sure that our services are as integrated as possible with the clinic … So it helps to break down the stigma that health workers might have towards … effective substance abuse treatment is part of health.’ (HCP 05)

#### Confidentiality, stigma and fear of judgement

ETAU participants and KPs expressed fear of stigma and judgement from HCPs, particularly when they missed substance use treatment programme sessions and HIV follow-up appointments. This anxiety often led to disengagement from the programme, driven by concerns that a single absence could result in exclusion or negative treatment. One ETAU participant said:

‘I was really scared because I had defaulted … I‘m scared of the reaction from them.’ (ETAU 1009)

The fear of discrimination was described in participant accounts of a lack of privacy. An ETAU participant reported his fear of attending a substance use treatment programme:

‘[*He*] didn’t know how these people [*healthcare providers*] were going to react or judge.’ (ETAU 1042)

HCPs also highlighted the lack of confidentiality at facilities as a barrier, particularly because of stigma. One explained that once the purpose of the visit is known:

‘[*S*]ome of them [*patients*] would have a problem with being seen by a person going there [*substance use treatment programmes*].’ (HCP 02)

A KP defaulted on treatment through fear that attending the clinic with her partner would reveal her HIV status:

‘I did not want him [*her partner*] to find out what is it exactly that I was doing at the clinic, so I ended up not completing my sessions.’ (KP 1001)

In contrast, confidentiality encouraged engagement: one KP noted that their information had been de-identified:

‘[*T*]he information that I give you will be attached to the number.’ (KP 1025)

#### Therapeutic relationships and provider capacity

Most KPs reported that positive interactions with HCPs supported their treatment adherence greatly. Strong therapeutic relationships characterised by a caring, non-judgemental approach and personalised follow-up, motivated continued engagement. All participants valued counselling, including aspects of motivational interviewing which encouraged them to stay in the programme.

A KP described the positive relationship she had with the healthcare worker:

‘I was talking to her as if I was talking to a friend.’ (KP 1018)

Another KP said if she missed a session, the health care worker:

‘[*W*]ould call me and check up on me … You know, there’s, there’s a difference between someone who is curious and someone who is concerned.’ (KP 1018)

Healthcare providers highlighted multiple challenges affecting their capacity to deliver effective services. One provider noted that training on substance use remains insufficient across the healthcare workforce. Providers also reported limited knowledge of referral pathways and available substance use treatment programmes, which hindered appropriate patient referrals and contributed to underutilisation of these services. They suggested that enhancing staff education might help to reduce stigma and strengthen service provision, stating:

‘[*Healthcare providers*] can learn not to judge. … We need to be educated on how to accept people the way they are.’ (HCP 03)

Another HCP reported:

‘[*S*]hortage of staff is really killing the morale of the staff as well.’ (HCP 01)

They noted that increased staff could assist with identifying issues and screening of patients:

‘[*C*]an be able to identify people with problems. So, I think having more staff will eliminate the barriers. And it will, it will help everyone.’ (HCP 01)

### Readiness to change

Most patient participants highlighted several factors that facilitated their readiness to change, including the negative physical effects of substance use, their sense of responsibility towards their families, interpersonal conflicts, and a strong intrinsic motivation to change. These factors served as key motivators for their commitment to the treatment programmes.

One KP explained that their motivation was due to increased awareness into his substance use:

‘It’s knowing that I had a problem. I had a problem with alcohol. I had a problem with adherence. And I knew that, you know, if, if I’m coming here, I’m coming to get help. I knew that was what I was going to benefit from coming here.’ (KP 1037)

An ETAU participant described the effect of personal motivation:

‘Because I wanted to change. It’s me taking life seriously now and wanting to get a job and wanting to be busy and telling myself that I should go and, you know, look for a job and you know, just change my life.’ (ETAU 1055)

#### Family responsibilities

The responsibility of childcare, in particular, was mentioned by a few participants and was associated with an increased readiness to change. One patient reported that her partner made her aware that she was neglecting her child – the KP noted:

‘I can say he [*partner*] played a positive role because he is someone who would actually tell me that “[*participant’s name*], can you please stop drinking alcohol? Please just be a mother for once because each and every weekend, you are always out with your friends.”’ (KP 1037)

Another ETAU participant reported:

‘[*B*]ecause of my drinking alcohol, I lost a lot of things. Now my child is sitting at home; she does not get the grant money because of my mistakes. So, I had to sit down and really think a lot about how I am drinking alcohol because it is my fault. It’s because of my lifestyle.’ (ETAU 1004)

#### Role of psychoeducation and supportive interactions

Psychoeducation was identified as a key facilitator of change, helping participants to understand the risks of substance use and the importance of adherence. Most participants emphasised that gaining insight into the harmful effects of substance use and non-adherence to treatment motivated patients to prioritise their health, remain engaged in care, and pursue recovery – particularly among those in the Project Khanya intervention. This newfound understanding played a crucial role in motivating them to stay engaged in the programme and to work towards recovery. One HCP participant reported, to encourage substance use cessation, ETAU participants and KPs:

‘[*W*]ould need to be taught about the dangers of drugs, how dangerous drugs are.’ (HCP 08)

A KP stated that they were unaware of the extent of their substance use until engaging in the substance use treatment programme:

‘[*The Khanya intervention*] brought to my attention that I’m drinking a lot and I should reduce alcohol, you know.’ (KP1001)

An ETAU participant shared the same sentiment:

‘What has helped me change is coming to you. You know, after coming to you, I realised that I need to change, and so I did.’ (ETAU 1004)

A KP noted that a good therapeutic relationship and staff interactions facilitated his ongoing engagement in care and assisted with his adherence:

‘I think it’s the encouragement that I received … I decided to take the information that they were giving me more seriously and it really helped me.’ (KP 1065)

### Financial constraints

#### Unemployment and competing demands

The majority of participants identified that unemployment can contribute to risky behaviours, including increased substance use, crime and negative health implications including non-adherence to ART.

One HCP observed:

‘It is unemployment … they [*people with HIV*] will start to derail from the norm and then engage in, in activities that are not helpful, forgetting that some of them are taking treatment, the priority will be pleasing the group more than their health.’ (HCP 01)

On the other hand, both ETAU participants and KPs identified work obligations, driven by the need to meet family responsibilities, often prioritising employment over treatment sessions. A KP explained:

‘I‘m self-employed; it did kind of get in the way of having to come to the sessions because I had to get jobs that I need to quickly take them because I need to get money.’ (KP 1063).

A HCP corroborated the above:

‘Clients are maybe finding informal employment, for this week, or for this day, and that obviously impacts attendance.’ (HCP 05)

#### Transportation barriers

Transportation costs were significant barriers to attending substance use treatment programmes. Many ETAU participants and KPs could not afford the cost of public transport to attend sessions, leading them to miss sessions or to avoid starting treatment altogether. One of the ETAU participant, who is unemployed, said:

‘I won‘t be able to attend those sessions. And also, I did not have, you know, transport money to go to the Matrix.’ (ETAU 1004)

A healthcare worker also reported:

‘Sometimes it’s a financial issue. Clients can’t afford taxi fares.’ (HCP 05)

#### Facilitating effects of incentives

Financial incentives, such as food vouchers, transportation assistance and food parcels were recognised as important facilitators for consistent attendance and addressed the issue of transportation and accessibility challenges. These incentives helped to alleviate financial burdens and encouraged programme participation.

A HCP, who is part of Project Khanya, noted that incentives helped to ensure attendance at their programme:

‘[*T*]hey are getting an incentive because you must understand that … at our facility where we are situated, the people that are staying they are really people that do not have anything.’ (HCP 03)

Another HCP said:

‘They’d [*patients*] be really be excited to come and attend to your, to your interventions because they are getting an incentive … it assists them so that they can get something to eat, so for them to take their treatment.’ (HCP 03)

## Discussion

Across participant and HCP narratives, the major influences on health behaviour and treatment outcomes were identified as: the positive and negative impact of social support, the pervasive effects of substances on a person’s relationships and health, varying readiness to change, the benefits of psychoeducation, and persistent financial challenges.

Social support emerged as a key facilitator for engagement in both HIV and substance use treatment, with participants and HCPs emphasising the profound importance of family, partners, and community in promoting adherence. Participants frequently highlighted practical assistance and emotional encouragement, corroborating existing evidence that stable support systems improve health outcomes significantly.^12^ However, the social environment also posed significant challenges. Peer pressure and community stigma often encouraged substance use and discouraged treatment engagement.^29^ Consequently, our findings suggest that effective interventions could incorporate family involvement proactively and address counterproductive peer and community dynamics directly.

The quality of the therapeutic relationship proved to be a pivotal and often determining factor influencing the engagement of participants with healthcare services. Participants who interacted with empathetic, confidential, and non-judgemental HIV care and substance use providers were more likely to establish trust and to demonstrate adherence to treatment. Conversely, stigma exhibited by healthcare professionals contributed to reduced health-seeking behaviours and demonstrable poor adherence among participants. This was particularly evident in the context of intersecting stigmas related to both HIV and substance use, reflecting the patterns of enacted and internalised stigma documented in prior research.^16,19^ Such stigma often stemmed from insufficient provider training, limited understanding of SUDs, and systemic staffing constraints, all of which served to exacerbate patient disengagement.^22^ These findings highlight the imperative for targeted interventions that foster stronger provider–patient relationships.

Ongoing substance use emerged as a complex and persistent multifaceted barrier, impeding both treatment adherence to ART and substance use treatment programmes for participants significantly. Both participants and providers described its negative impact on medication adherence, clinic attendance, and ongoing participation in health services. Substance use was also seen as a coping mechanism for psychosocial distress linked to stigma, trauma, poverty, and the challenges of living with chronic conditions like HIV.^19^ While periods of reduction of substance use improved health outcomes, relapse often led to disengagement from care. Given the complex social factors that influence substance use in LMICs, further research is needed to guide the development of multi-level contextually appropriate and comprehensive interventions.

Healthcare providers indicated that addressing ongoing substance use effectively requires insight and readiness to change from participants. These are influenced by a combination of internal motivations, such as personal insight, a desire for lifestyle improvement, and self-efficacy as well as external responsibilities, including family roles and caregiving pressures.^15,19^ However, for some, the initial reduction of substance use led to disengagement from treatment programmes, as they perceived continued care to be unnecessary. These findings underscore the necessity of sustained investment in capacity building and the expansion of outreach to improve early engagement and access to substance use treatment.

Psychoeducation and increased awareness of the negative effects of substance use were identified as key facilitators of engagement. Gaining insight into the risks of substance use and the benefits of adherence motivated participants to prioritise their health. The development of trust and rapport with HCPs amplified the impact of psychoeducation, reinforcing motivation to change. However, limited awareness of treatment options, especially in under-resourced settings, and inconsistent provider training, hindered the delivery of effective psychoeducational interventions.^22^ Both participants and providers emphasised the urgent need for enhanced staff training aimed at reducing stigma, improving referral pathways, and fostering supportive clinical environments.

Community outreach, led by both lay and trained healthcare workers, plays a crucial role in improving access to substance use treatment by extending care beyond traditional clinical settings.^35^ These efforts foster culturally sensitive, stigma-reducing environments and address practical barriers such as accessibility and continuity of care.^22,27^ Both providers and patients valued integrated, community-based models for their ability to alleviate facility congestion from clinics, reach underserved populations and provide education and awareness to communities in a culturally sensitive manner to address issues of stigma.^17^ However, concerns persist about privacy and provider stigma, underscoring the need for sustained investment in capacity building and decentralised, culturally tailored interventions that reflect local realities and promote long-term engagement. These findings support global recommendations advocating decentralised, integrated service delivery in LMICs. Therefore, future interventions should prioritise tailored, culturally sensitive, and community-driven approaches that reflect the contextual realities of the populations served.

In the South African LMIC context, financial constraints emerged as a major barrier to care engagement.^17^ Unemployment increased psychosocial stress and substance use, while employment often led participants to prioritise income over treatment attendance. Transportation costs and resource scarcity limited access further. Both patients and providers emphasised that incentives such as food parcels or transport assistance were crucial in encouraging consistent participation. This finding was supported by literature stating that incentives improved access to HIV care.^36^ However, most existing research originates from middle- to high-income countries, often overlooking the impact of poverty and unemployment.^23^ A pressing need exists for future research, specifically exploring these complex systemic barriers in LMICs to inform more equitable and effective interventions.^23^

Collectively, these findings highlight the need for multi-level interventions for SUD that address not only individual behaviour but also the social, structural, and systemic barriers to substance use treatment engagement. Project Khanya aimed to address the challenges faced by people with HIV and SUDs, focusing on factors that enhanced their engagement in care. The success of Project Khanya provides evidence that addressing these factors can improve both patient satisfaction and health outcomes. Narratives from patients and HCPs underscore the importance of holistic, integrated, and person-centred approaches that acknowledge the interconnected realities of HIV, substance use, and the broader environments in which people live. Strengthening therapeutic relationships, promoting family and community involvement, reducing stigma, and alleviating economic hardship are essential components for improving adherence and achieving sustainable health outcomes in this population.

### Limitations and future research

The findings of this study are specific to a LMIC community and may not be generalisable across different regions or populations. As a secondary qualitative analysis, the scope and depth of insights were restricted. Limited demographic information on participants and HCPs, as well as details on substance types and interview selection, further constrained the findings. A limited number of interviews were made available for analysis, which does not align with the CORE-Q requirements that affect data saturation. Owing to time constraints during the data analysis, the codebook developed by the first coder was not modified by the second coder, thereby limiting the insight and variety of identified codes. Intercoder reliability was not formally assessed, as both coders adhered to the shared codebook. Further research is needed to explore longitudinal outcomes of integrated interventions like Project Khanya, particularly regarding long-term reduction of substance use and sustained treatment adherence.

## Conclusion

This study examined the barriers and facilitators influencing engagement in substance use treatment programmes among people with HIV and SUDs. Substance use in this population is associated with poor adherence to ART, compromised health status, and exacerbation of both social and clinical challenges. Key barriers to sustained engagement in treatment include limited social support, pervasive stigma, structural and service-related inadequacies, financial limitations, and insufficient awareness of available services. Conversely, primary facilitators of engagement encompassed strong support from family and community networks, incentives provided by treatment programmes, assurance of privacy, and individual motivation for recovery. These findings offer insights that warrant further investigation and can inform the development of contextually appropriate interventions aimed at improving health and social outcomes in this high-risk population. Future research should include mixed-methods studies that combine quantitative data with qualitative insights to provide a more comprehensive understanding of engagement factors. Longitudinal research is also recommended to track participants over time and to examine how barriers and facilitators influence sustained engagement and treatment outcomes. In addition, studies conducted in other low- to middle-income countries are needed, as most existing research on attendance in substance use treatment programmes has been carried out in high-income settings.

## References

[1] Statistics South Africa. Mid-year population estimates: 28 July 2022. Statistical release P0302 [homepage on the Internet]. Statistics South Africa; 2022 [cited 2024 Mar 17]. Available from: https://www.statssa.gov.za/publications/P0302/P03022022.pdf

[2] Visagie J, Posel D. A reconsideration of what and who is middle class in South Africa. Dev South Afr. 2013;30(2):149–167. 10.1080/0376835X.2013.797224

[3] Zuma K, Simbayi L, Zungu N, et al. The HIV epidemic in South Africa: Key findings from 2017 national population-based survey. IJERPH. 2022;19(13): 8125. 10.3390/ijerph19138125 35805784 PMC9265818

[4] Asamoah-Odei E, Calleja JM, Boerma JT. HIV prevalence and trends in sub-Saharan Africa: No decline and large subregional differences. Lancet. 2004;364(9428): 35–40. 10.1016/S0140-6736(04)16587-2 15234854

[5] Cain D, Pitpitan EV, Eaton L, et al. Collective efficacy and HIV prevention in South African townships. J Community Health. 2013;38(5):885–193. 10.1007/s10900-013-9694-9 23660646 PMC3769453

[6] Kalichman SC, Simbayi LC, Kagee A, et al. Associations of poverty, substance use, and HIV transmission risk behaviors in three South African communities. Soc Sci Med. 2006;62(7):1641–1649. 10.1016/j.socscimed.2005.08.021 16213078

[7] Herman AA, Stein DJ, Seedat S, Heeringa SG, Moomal H, Williams DR. The South African Stress and Health (SASH) study: 12-month and lifetime prevalence of common mental disorders. SAMJ. 2009 [cited 2024 Mar 17];99(5):339–344. Available from: https://www.ajol.info/index.php/samj/article/view/50764 19588796 PMC3191537

[8] Shisana O, Rehle T, Simbayi LC, et al. D. South African national HIV prevalence, incidence and behaviour survey [homepage on the Internet]. 2012 [cited 2024 Mar 24]. Available from: http://hdl.handle.net/20.500.11910/2490

[9] World Health Organization. The Alcohol, Smoking and Substance Involvement Screening Test (ASSIST): Development, reliability and feasibility [homepage on the Internet]. World Health Organization; 2002 [cited 2025 Jul 6]. https://www.who.int/publications/i/item/978924159938-2

[10] Barnard I. Psychosocial profile of personality traits self-regulation and substance abuse tendencies of adolescents in Gauteng: Efficacy of a competency skills programme [doctoral dissertation]. North-West University; 2014.

[11] Browne FA, Wechsberg WM. The intersecting risks of substance use and HIV risk among substance-using South African men and women. Curr Opin Psychiatry. 2010;23(3):205–209. 10.1097/YCO.0b013e32833864eb 20308902 PMC3784346

[12] Farhoudian A, Razaghi E, Hooshyari Z, et al. Barriers and facilitators to substance use disorder treatment: An overview of systematic reviews. J Subs Abuse Treat. 2022;16:11782218221118462. 10.1177/11782218221118462 PMC9434658 36062252

[13] Kuchinad KE, Hutton HE, Monroe AK, Anderson G, Moore RD, Chander G. A qualitative study of barriers to and facilitators of optimal engagement in care among PLWH and substance use/misuse. BMC Res Notes. 2016;9(1):229. 10.1186/s13104-016-2032-4 27103162 PMC4841053

[14] Belus JM, Regenauer KS, Hutman E, et al. Substance use referral, treatment utilization, and patient costs associated with problematic substance use in people living with HIV in Cape Town, South Africa. Drug Alcohol Depend Rep. 2022;2:100035. 10.1016/j.dadr.2022.100035 36845899 PMC9948858

[15] Browne T, Priester MA, Clone S, Iachini A, DeHart D, Hock R. Barriers and facilitators to substance use treatment in the rural south: A qualitative study. J Rural Health. 2016;32(1):92–101. 10.1111/jrh.12129 26184098

[16] Kulesza M, Larimer ME, Rao D. Substance use related stigma: What we know and the way forward. J Addict Behav Ther Rehabil. 2013 27;2(2):782. 10.4172/2324-9005.1000106 25401117 PMC4228689

[17] Magidson JF, Joska JA, Regenauer KS, et al. ‘Someone who is in this thing that I am suffering from’: The role of peers and other facilitators for task sharing substance use treatment in South African HIV care. Int J Drug Policy. 2019;70:61–69. 10.1016/j.drugpo.2018.11.004 31082664 PMC6679990

[18] Myers B, Parry CD. Access to substance abuse treatment services for black South Africans: Findings from audits of specialist treatment facilities in Cape Town and Gauteng. Afr J Psychiatry. 2005;8(1):15–19. 10.4314/ajpsy.v8i1.30179

[19] Regenauer KS, Myers B, Batchelder AW, Magidson JF. ‘That person stopped being human’: Intersecting HIV and substance use stigma among patients and providers in South Africa. Drug Alcohol Depend. 2020;216:108322. 10.1016/j.drugalcdep.2020.108322 33010712 PMC7673102

[20] Wogen J, Restrepo MT. Human rights, stigma, and substance use. HHR. 2020 [cited 2024 Mar 24];22(1):51. Available from: https://pmc.ncbi.nlm.nih.gov/articles/PMC7348456/PMC7348456 32669788

[21] Myers B, Sorsdahl K. Addressing substance use within primary health care settings in South Africa: Opportunities and challenges. Addicta Turk J Addict. 2014;1(2): 80–94. 10.15805/addicta.2014.1.2.023

[22] Myers BJ, Louw J, Pasche SC. Inequitable access to substance abuse treatment services in Cape Town, South Africa. Subst Abuse Treat Prevent Policy. 2010;5(1):28. 10.1186/1747-597X-5-28 PMC2992042 21073759

[23] Haldane V, Cervero-Liceras F, Chuah FL, et al. Integrating HIV and substance use services: A systematic review. J Int. AIDS Soc. 2017;20(1):21585. 10.7448/IAS.20.1.21585 28692211 PMC5515016

[24] Turan JM, Elafros MA, Logie CH, Banik S, et al. Challenges and opportunities in examining and addressing intersectional stigma and health. Focus. 2025;23(1): 70–84. 10.1176/appi.focus.25023006 39776461 PMC11701822

[25] Gonzalez A, Barinas J, O’Cleirigh C. Substance use: Impact on adherence and HIV medical treatment. Curr HIV/AIDS Rep. 2011;8(4):223–34. 10.1007/s11904-011-0093-5 21858414

[26] Petoumenos K, Law MG. Smoking, alcohol and illicit drug use effects on survival in HIV-positive persons. Curr Opin HIV AIDS. 2016;11(5):514–520. 10.1097/COH.0000000000000306 27327615

[27] Rose AL, Belus JM, Hines AC, et al. SA. Patient and provider perceptions of a peer-delivered intervention (‘Khanya’) to improve anti-retroviral adherence and substance use in South Africa: A mixed methods analysis. Glob Ment Health. 2022;9:439–447. 10.1017/gmh.2022.47 PMC9807005 36618732

[28] Magidson JF, Joska JA, Belus JM, et al. Project Khanya: Results from a pilot randomized type 1 hybrid effectiveness-implementation trial of a peer-delivered behavioural intervention for ART adherence and substance use in HIV care in South Africa. J Int. AIDS Soc. 2021;24:e25720. 10.1002/jia2.25720 34164935 PMC8222840

[29] Belus JM, Rose AL, Andersen LS, et al. Adapting a behavioral intervention for alcohol use and HIV medication adherence for lay counselor delivery in Cape Town, South Africa: A case series. Cogn Behav Pract. 2022;29(2):454–467. 10.1016/j.cbpra.2020.10.003 36171964 PMC9512118

[30] City of Cape Town. Help and treatment for your addiction [homepage on the Internet]. Cape Town: City of Cape Town; 2021 [cited 2025 Sep 27]. Available from: https://www.capetown.gov.za/.com

[31] Braun V, Clarke V. Using thematic analysis in psychology. Qual Res Psychol. 2006;3(2):77–101. 10.1191/1478088706qp063oa

[32] Azeem M, Salfi NA, Dogar AH. Usage of NVivo software for qualitative data analysis. AR Int. 2012;2(1):262–266.

[33] Ahmed SK. Sample size for saturation in qualitative research: Debates, definitions, and strategies. J Med Surg Publ Health. 2025;5:100171. 10.1016/j.glmedi.2024.100171

[34] Hennink M, Kaiser, BN. Sample sizes for saturation in qualitative research: A systematic review of empirical tests. Soc Sci Med. 2022;292:114523. 10.1016/j.socscimed.2021.114523 34785096

[35] Odo F. The impact of outreach work, interventions and health outcomes in multicultural communities – A literature review [Bachelor’s thesis]. Diaconia University of Applied Sciences; 2025 [cited 2025 July 06]. Available from: https://www.theseus.fi/handle/10024/895838

[36] Schlehr SR, Singh L, Nyatela A, Nqakala S, Lalla-Edward ST. Experiences in receiving financial incentives to access HIV care in Johannesburg, South Africa. South Afr J HIV Med. 2022;23(1): 1426. 10.4102/sajhivmed.v23i1.1426 36479419 PMC9724036

